# Dengue fever and dengue haemorrhagic fever in adolescents and adults

**DOI:** 10.1179/2046904712Z.00000000049

**Published:** 2012-05

**Authors:** Terapong Tantawichien

**Affiliations:** Division of Infectious Diseases, Department of Medicine, Faculty of Medicine, Chulalongkorn University, Bangkok, Thailand

**Keywords:** Dengue, Adults, Adolescents, DHF, Dengue haemorrhagic fever, Age

## Abstract

Dengue fever (DF) is endemic in tropical and subtropical zones and the prevalence is increasing across South-east Asia, Africa, the Western Pacific and the Americas. In recent years, the spread of unplanned urbanisation, with associated substandard housing, overcrowding and deterioration in water, sewage and waste management systems, has created ideal conditions for increased transmission of the dengue virus in tropical urban centres. While dengue infection has traditionally been considered a paediatric disease, the age distribution of dengue has been rising and more cases have been observed in adolescents and adults. Furthermore, the development of tourism in the tropics has led to an increase in the number of tourists who become infected, most of whom are adults. Symptoms and risk factors for dengue haemorrhagic fever (DHF) and severe dengue differ between children and adults, with co-morbidities and incidence in more elderly patients associated with greater risk of mortality. Treatment options for DF and DHF in adults, as for children, centre round fluid replacement (either orally or intravenously, depending on severity) and antipyretics. Further data are needed on the optimal treatment of adult patients.

## Background

In hyperendemic areas in Asia, dengue fever (DF) and dengue haemorrhagic fever (DHF) affect mainly children under 15 years of age.^1^ The age distribution is different in the Americas where these syndromes occur in all age groups, although the majority of fatalities during epidemics occur in children.^2^ This article discusses the impact of dengue in adolescents and adults.

## Dengue in Different Age Groups

Data from several South-east Asian countries have shown that the average/mean age of reported dengue cases has increased from 5–9 years to older children and adults.^3^–^7^ In Thailand, affected adults over 15 years of age comprise 30–40% of dengue cases. At present, the morbidity rate of DHF is declining in Thailand, while the average age of patients with dengue infection is increasing.8,^9^ Dengue infection in adolescents and adults is also a potential hazard in international travellers returning from endemic areas, especially South-east Asia,^10^–^13^ a topic covered in more detail in the Wilder-Smith article ‘Dengue infections in travellers’ in this supplement.^14^

Dengue virus infection produces a spectrum of clinical illness ranging through undifferentiated fever, DF and self-limiting febrile illness associated with headache, myalgia and thrombocytopenia. DHF and dengue shock syndrome (DSS) are more serious and can be fatal.15,^16^ The classification of dengue fever severity is explored in the Hadinegoro article ‘The revised WHO dengue case classification: does the new system need to be modified?’, also in this supplement.^17^

Several factors may influence disease severity, including host factors, virus serotype or genotype, sequence of virus infection, differences in dengue cross-reactive antibody, and T-cell responses.^18^ DF is usually self-limiting, and death is uncommon. However, age-related differences in dengue severity are poorly understood and data on clinical features in adult patients are limited.^3^–^5^ Older age has previously been reported to be a risk factor for mortality in patients with DF or DHF as the co-morbidities associated with ageing and waning immunity pose a substantial risk for fatality in elderly patients with active infection.19,^20^ Although shock and plasma leakage seem to be more prevalent in younger patients, the frequency of internal haemorrhage augments as age increases.^21^ Furthermore, complications of dengue infection observed in adults, including DF with unusual bleeding and DHF, have been increasing.^22^–^24^

## Clinical Manifestations

The clinical characteristics in 140 adults infected with dengue virus during the Bangkok dengue epidemic in 1997–1998 are summarised in Table 1.

**Table 1:** The clinical manifestations of DF in 140 adults during the Bangkok epidemic in 1997–1998

	DF/DHF	DF	DHF
	*n* = 140	*n* = 89	*n* = 51
Age, y, mean, [range]	26·9 [15–67]	28·6 [15–67]	23·4 [15–44]
Total duration of fever, d, mean, [range]	5·2 [2–8]	5·2 [2–8]	5·2 [3–8]
Fever, %	100	100	100
Nausea/vomiting, %	47·1	40·4	58·8
Headache, %	37·8	38·2	37·3
Diarrhoea, %	25·0	33·3	21·2
Myalgia, %	25·7	25·8	25·6
Abdominal pain, %	23·6	12·3	43·1
Haemorrhagic manifestations, %	35·7	24·7	54·9
Petechiae	22·1	14·6	35·2
Epistaxis	7·8	4·4	14·3
Gum bleeding	7·1	5·5	10·2
Haematemesis	2·1	0	5·9
Vaginal bleeding	24·6	21	31·6
Bleeding >2 sites	27	6·7	19·6
Rash (occurring in convalescence), %	27·8 (5)	31·5	21·6
Hypotension/pulse pressure <20 mmHg, %	2·1	0	5·8
Jaundice, %	0·7	0	1·9
Epigastric or RUQ tenderness, %	18·6	10·1	29·4
Hepatomegaly, %	21·4	11·2	39·2
Splenomegaly (by ultrasonography), %	2·1	0	5·9
Ascites (by ultrasonography), %	3·6	0	9·8
Pleural effusion (by chest radiograph), %	10·7	0	29·4

RUQ, right upper quadrant

## Manifestations of Dengue Fever

During the acute febrile phase of DF, usually lasting 3–8 days, many of the clinical symptoms resemble those of DHF, including fever, nausea/vomiting, headache, rash and myalgia; abdominal pain and severe or widespread bleeding are less frequent in DF.^25^ Minor haemorrhagic manifestations such as petechiae, epistaxis and gingival bleeding do sometimes occur in DF, although they are rarely associated with severe haemorrhage leading to shock.^26^

## Manifestations of Dengue Haemorrhagic Fever

Owing to differences in capillary permeability, adults may be at lower risk than children of developing DHF.^27^ DHF can be distinguished from DF by the presence of increased vascular permeability (plasma leakage syndrome) and marked thrombocytopenia (<100,000/μl) associated with bleeding, hepatomegaly and/or abnormal liver function. Acute respiratory failure, although a rare complication in adults, has a high mortality rate.^28^ Although children are more likely to develop hypovolaemic shock characterised by increased microvascular permeability in DHF, a high mortality rate has been observed in adult patients.^29^ The outcome of DHF and DSS depends largely on early diagnosis and the immediate replacement of fluid.

Haemorrhage contributes to dengue morbidity and mortality, especially during the severe thrombocytopenia and toxic haemorrhagic stage (3–5 days after illness onset).^30^ In Thailand, bleeding manifestations including petechiae, epistaxis and menorrhagia have been observed frequently in adults with DF or DHF (own data, Table 1), although upper gastro-intestinal (GI) bleeding is the most common type of severe haemorrhage.^30^ In reports of endoscopic examination of dengue patients with upper GI tract bleeding, haemorrhagic gastritis was the most common finding (40·9–58·5%).^31^–^33^ However, massive haematemesis may occur in adults with DF or DHF owing to peptic ulcers, which is not associated with profound shock, as in previous reports in children.^33^ In patients with pre-existing peptic ulcers, severe or even fatal bleeding can be precipitated by dengue infection, though in most cases supportive therapy and blood transfusions are adequate to manage this complication.^33^ Subcapsular splenic bleeding and rupture have also been reported in adults with dengue infection.^34,35^ However, splenic rupture in patients with haemorrhagic dengue is uncommon and can happen spontaneously or as a result of trauma, which may be minor or unnoticed.^34,35^

Menorrhagia is common in female adults with DF/DHF (up to 25%).^7^ Uterine haemorrhage resulting in spontaneous abortion, premature labour and severe postpartum bleeding has been observed in women with DF/DHF.^36^–^38^ In patients with dengue during onset of labour, blood or platelet transfusion may be required in cases with severe bleeding or where caesarean section is required.38,^39^

Increased liver enzymes [alanine aminotransferase (ALT) and aspartate aminotransferase (AST)] have been found in children and adults during dengue infection, indicating liver involvement.^40^–^42^ Unlike conventional viral hepatitis, AST level is higher than ALT in dengue infection,^41^ with levels increasing to a maximum 7–9 days after onset of symptoms, decreasing to normal within 2 weeks.^41^ Pre-existing liver disease such as chronic hepatitis and haemoglobinopathies are more likely to be present in adults than in children with dengue and may aggravate the liver impairment.19,^43^ Liver injury is often self-limiting, but fulminant hepatitis and death have been reported.42,^44^ The association between severe liver disease and encephalopathy is well described in children and adults with DF/DHF, and high mortality has been reported in dengue patients with hepatitis and encephalopathy.^45^

More unusual manifestations of dengue infection in adults include severe internal haemorrhage, cardiomyopathy, cardiac arrhythmias, adult respiratory distress syndrome (ARDS), rhabdomyolysis, pancreatitis, appendicitis, co-infection with other tropical diseases, and neurological phenomena such as altered consciousness, seizures and coma owing to encephalitis and encephalopathy.^46^–^53^ Neurological manifestations secondary to dengue infection were ascribed to non-specific complications such as myelitis, optic neuritis, polyradiculopathy or neuropathy.54,^55^ Possible causes of dengue encephalopathy include hypotension, cerebral oedema, focal haemorrhage, hyponatraemia and fulminant hepatic failure.51,55,^56^ However, a recently documented possibility is dengue invasion of the nervous system.57,^58^ Furthermore, some studies have indicated that 5·5% of patients with DHF have dual infections such as urinary tract infection, diarrhoea or bacteraemia.^19^ Failure to correctly diagnose any concurrent infection in patients with DHF may lead to greater morbidity or mortality, which would otherwise be preventable. Prolonged fever and acute renal failure are independent predictive factors for dual infection.^19^

## Treatment of Dengue Infection in Adults

**Figure 1:**
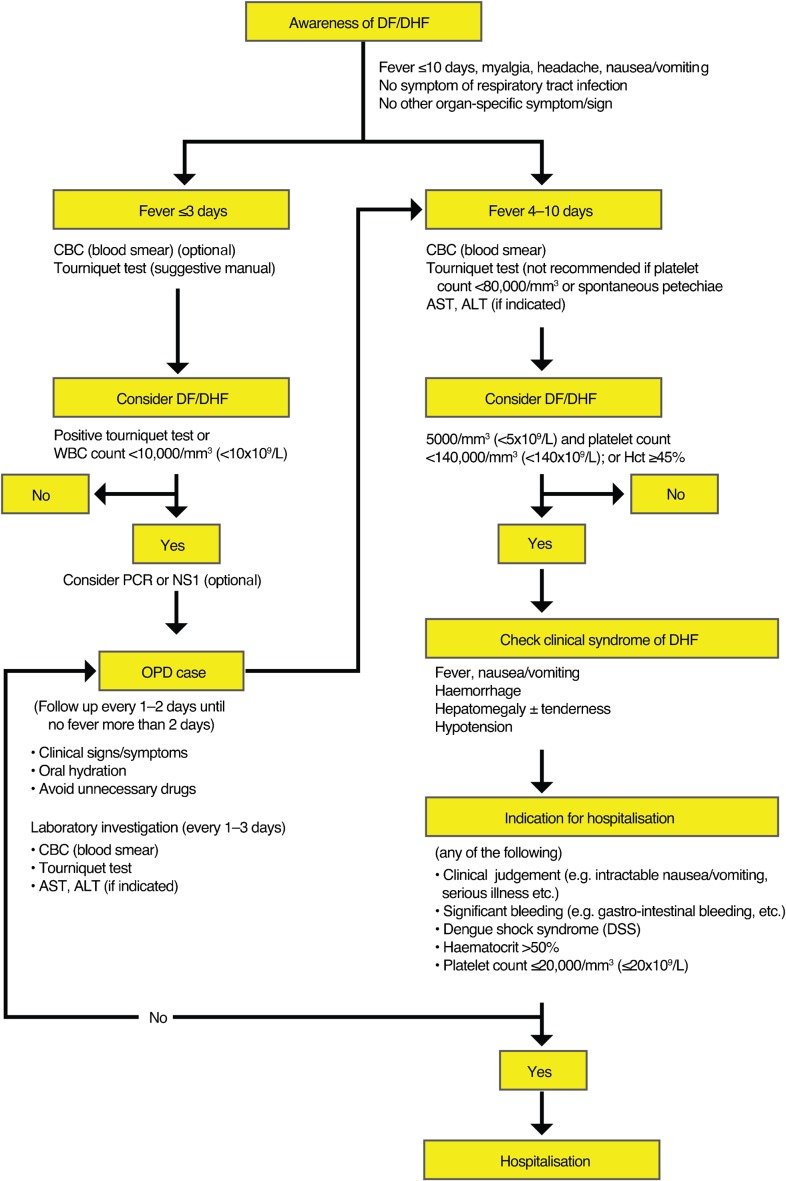
Management of adult dengue in Thailand (clinical practice guidelines by the Infectious Diseases Society of Thailand, 2006)

**Figure 2:**
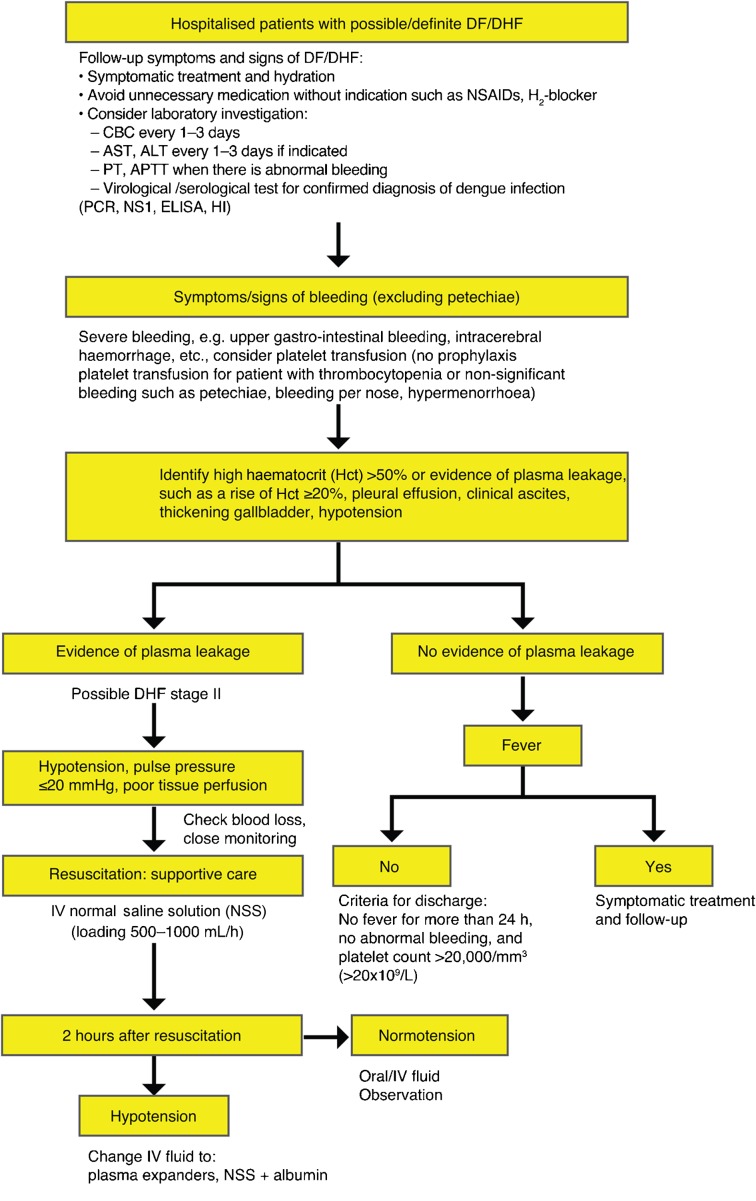
Management of adult dengue in Thailand, continued (clinical practice guidelines by the Infectious Diseases Society of Thailand, 2006)

Currently, there are no specific therapeutic agents for dengue. In adults, early recognition of dengue infection, bleeding and signs of circulatory collapse reduces mortality with dengue (Figs 1 and 2).

Mild dengue infection may be treated with oral hydration and antipyretics.^59^ Agents such as salicylates, non-steroidal anti-inflammatory drugs and traditional medicines that may have hepatotoxic effects must be avoided.^16^ Attentive clinical monitoring of patients with suspected DHF, along with anticipatory and supportive care, are life-saving and reduce fatality rates. To identify the need for intravenous fluid therapy, circulation and vascular leakage must be monitored by serial clinical assessments of pulse, blood pressure, skin perfusion, urine output and haematocrit.^16^ Patients with DHF need to be monitored closely for signs of shock for at least 24 hours after defervescence.^29^ Prompt fluid resuscitation remains the mainstay of treatment to counteract massive plasma leakage. In most adult cases, timely and effective intravenous crystalloid replacement of plasma losses results in a favourable outcome. If shock persists, immediate volume replacement with Ringer’s lactate, Ringer’s acetate or physiological saline should be followed by plasma or colloid solutions.^60^ Recently, three randomised controlled trials evaluated therapeutic responses to colloid and crystalloid solutions.^61^–^63^ Results indicate that Ringer’s lactate performed least well.^61^–^63^ Patients with a narrow pulse pressure (⩽10 mmHg), indicating more severe DSS, should benefit from initial resuscitation with colloid solution rather than crystalloid solution.^61^–^63^ Preventive transfusions may be harmful and should be avoided, and invasive procedures should be minimised to avoid haemorrhagic complications.

## Conclusion

Dengue infection is generally considered to be a paediatric disease but is currently a growing problem in adults throughout the tropics. Furthermore, dengue infection can be more severe in adults in whom early recognition of dengue infection, bleeding tendencies and signs of circulatory collapse would reduce mortality. Fluid replacement is the gold-standard therapeutic option for adults with dengue fever, as it is for children. However, further studies in adults are required to establish the best therapeutic approaches and determine whether any specific factors should be considered in terms of warning signs and risk factors.
